# Supplementary material for: ASV vs OTUs clustering: Effects on alpha, beta, and gamma diversities in microbiome metabarcoding studies
Source: PLoS One. 2024 Oct 3;19(10):e0309065. doi: 10.1371/journal.pone.0309065 (PMC11449282; doi:10.1371/journal.pone.0309065)
Supplement: S1 Fig — Boxed cells with grey background indicate significant differences for p<0.05. Abund: sequence reads abundance; ASV, O99, O97: number of different taxa resulting from the full ASV analysis or from the OTU clustering at 99% or 97% shared homology, respectively. These three prefixes apply for the remaining correlation indexes abbreviations whose suffixes indicate the following: BgPk: Berger-Parker Dominance; Brill: Brillouin Diversity Index; Equ: Equitability J; Ev: Community Evenness e^H/S; Fisha: Fisher alpha Diversity Index; Marg: Margalef Richness Index; Menh: Menhinick richness index; Sha: Shannon-Wiener H Diversity Index; Smp: Simpson 1-D Diversity Index. (DOCX) [file pone.0309065.s001.docx]

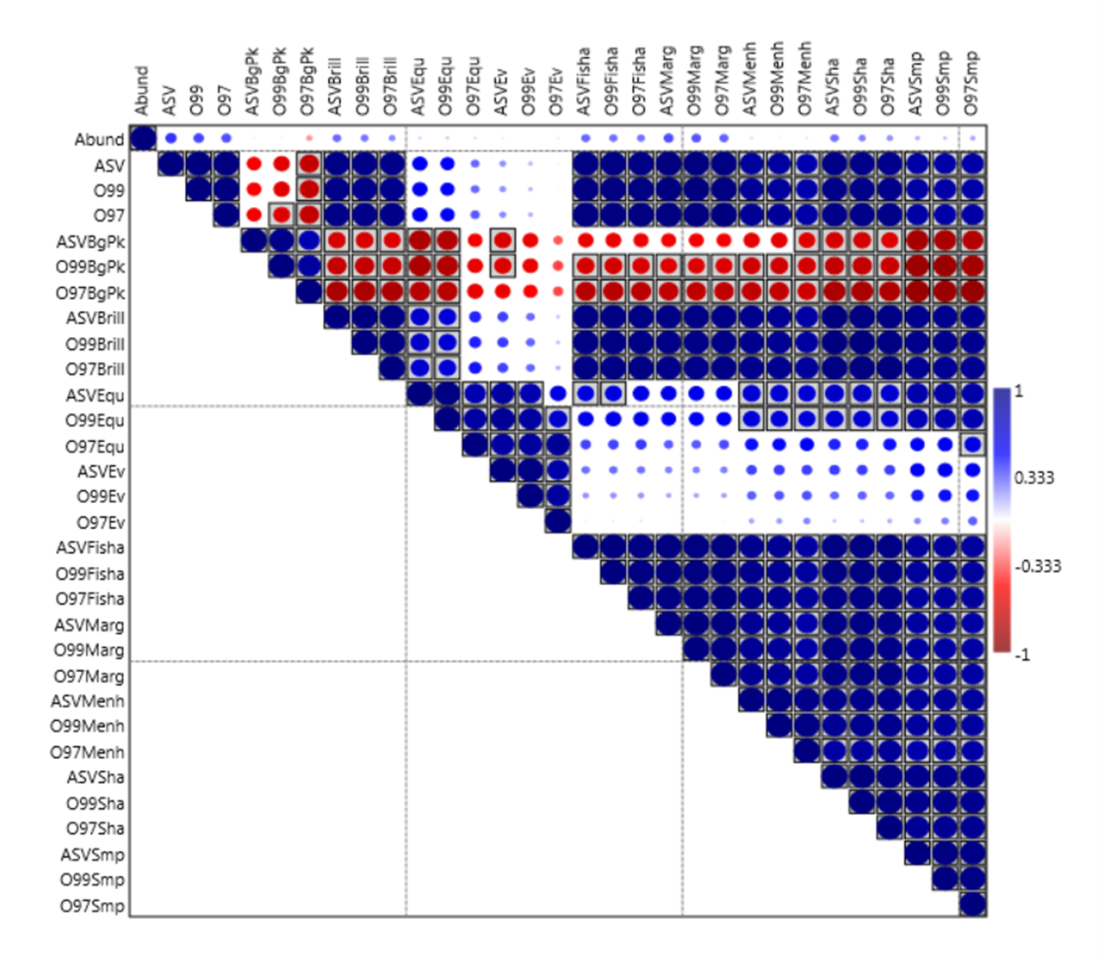


**Fig. S1. Correlation matrix (Spearman Rank Sum Coefficient with Bonferroni-corrected p values)** of the **pairwise comparisons across numbers of taxa and ecological indexes of the three sequence clustering approaches.** Boxed cells with grey background indicate significant differences for p<0.05. Abund: sequence reads abundance; ASV, O99, O97: number of different taxa resulting from the full ASV analysis or from the OTU clustering at 99% or 97 % shared homology, respectively. These three prefixes apply for the remaining correlation indexes abbreviations whose suffixes indicate the following: BgPk: Berger-Parker Dominance; Brill: Brillouin Diversity Index; Equ: Equitability J ; Ev: Community Evenness e^H/S; Fisha: Fisher alpha Diversity Index; Marg: Margalef Richness Index; Menh: Menhinick richness index; Sha: Shannon-Wiener H Diversity Index; Smp: Simpson 1-D Diversity Index.
