# Supplementary material for: ASV vs OTUs clustering: Effects on alpha, beta, and gamma diversities in microbiome metabarcoding studies
Source: PLoS One. 2024 Oct 3;19(10):e0309065. doi: 10.1371/journal.pone.0309065 (PMC11449282; doi:10.1371/journal.pone.0309065)
Supplement: S1 Text — (DOCX) [file pone.0309065.s003.docx]

**S1 Text. Bioinformatics Pipeline Scheme and command scripts**

#Import raw sequences to qiime environment

qiime tools import --type 'SampleData[SequencesWithQuality]' --input-path manifest.csv --output-path demux --input-format SingleEndFastqManifestPhred33

#summarize the import statistics

qiime demux summarize --i-data demux.qza --o-visualization demux

#Using cutadapt to trim the primers

qiime cutadapt trim-paired --i-demultiplexed-sequences demux.qza --p-front-f gtgycagcmgccgcggtaa --p-adapter-r ggactacnvgggtwtctaat --p-cores 20 --o-trimmed-sequences trimmed_demux.qza

#using qiime dada2 denoising to denoise and generate ASVs

qiime dada2 denoise-paired --o-table table.qza --o-representative-sequences rep_seqs.qza --o-denoising-stats denoise_stats --p-n-threads 25 --p-trunc-len-f 0 --p-trunc-len-r 0 --i-demultiplexed-seqs demux.qza

#tabulate denoising statistics

qiime metadata tabulate --m-input-file denoising_stats.qz.qza --o-visualization denoising_stats

#taxonomic classification of ASVs using Silva 138.1 classifier

qiime feature-classifier classify-sklearn --i-classifier silva-138-99.qza --i-reads rep_seqs.qza --o-classification taxonomy.qza

#using qiime vsearch to cluster the OTUs

qiime vsearch cluster-features-de-novo --i-table table.qza --i-sequences rep-seqs.qza --p-perc-identity 0.99 --o-clustered-table table-99.qza --o-clustered-sequences rep-seqs-99.qza

qiime vsearch cluster-features-de-novo --i-table table.qza --i-sequences rep-seqs.qza --p-perc-identity 0.97 --o-clustered-table table-97.qza --o-clustered-sequences rep-seqs-97.qza

#taxonomic classification of 99% OTUs using Silva 138.1 classifier

qiime feature-classifier classify-sklearn --i-classifier silva-138-99.qza --i-reads rep-seqs-99 --o-classification taxonomy.99.qza

qiime feature-classifier classify-sklearn --i-classifier silva-138-99.qza --i-reads rep-seqs-99 --o-classification taxonomy.97.qza

#exporting ASV and OTU tables

qiime tools export --input-path table.qza --output-path table_ASV

qiime tools export --input-path table-97.qza --output-path table_OTU.97

qiime tools export --input-path table-99.qza --output-path table_OTU.97

#converting biom tables to TSV format

biom convert -i table_ASV/feature-table.biom -o table_ASV.tsv --to-tsv

biom convert -i table_OTU.97/feature-table.biom -o table_OTU.97.tsv --to-tsv

biom convert -i table_OTU.97feature-table.biom -o table_OTU.97.tsv --to-tsv

#exporting taxonomy tables

qiime tools export --input-path taxonomy.qza --output-path taxonomy.tsv

qiime tools export --input-path taxonomy.97.qza --output-path taxonomy.97.tsv

qiime tools export --input-path taxonomy.99.qza --output-path taxonomy.99.tsv
